# Pain Management Strategies and Adverse Effects of Opioids in Patients with Neurotrauma with Acute and Chronic Pain

**DOI:** 10.1177/08977151251365585

**Published:** 2025-08-19

**Authors:** Marie-Ève McGennis, Marc-Aurèle Gagnon, Jérôme Paquet, Alexis F. Turgeon, Tassia Macedo, Caroline Côté, Mwanack Kakule Matina, Michael Verret, Lynne Moore, Andréane Richard-Denis, Line Guénette, Léonie Archambault, Cécile Duval, Mélanie Bérubé

**Affiliations:** ^1^ Population Health and Optimal Practices Research Unit (Trauma–Emergency–Critical Care Medicine), CHU de Québec-Université Laval Research Center, Québec City, Canada.; ^2^ Faculty of Nursing, Université Laval, Québec City, Canada.; ^3^ Department of Neurosciences, Service of Neurosurgery, CHU de Québec-Université Laval, Québec City, Canada.; ^4^ Department of Surgery, Division of Neurosurgery, Faculty of Medicine, Université Laval, Québec City, Canada.; ^5^ Department of Anesthesiology and Critical Care Medicine, Faculty of Medicine, Université Laval, Québec City, Canada.; ^6^ Quebec Pain Research Network, Sherbrooke, Canada.; ^7^ Department of Social and Preventive Medicine, Faculty of Medicine, Université Laval, Québec City, Canada.; ^8^ Department of Physical Medicine and Rehabilitation, Centre intégré universitaire du Nord de l’île-de-Montréal, Montréal, Canada.; ^9^ Faculty of Pharmacy, Université Laval, Québec City, Canada.; ^10^ Centre intégré universitaire de santé et des services sociaux du Centre-Sud-de-l’île-de-Montréal, Institut universitaire sur les dépendances, Montréal, Canada.

**Keywords:** neurotrauma, opioids, pain, pain management strategies, spinal cord injury, traumatic brain injury

## Abstract

Pain is prevalent and a major source of disability after a traumatic brain injury (TBI) and a spinal cord injury (SCI). With a view of reducing the pain burden in neurotrauma, this study aimed to describe the use of pain management strategies and the adverse effects of opioids in patients with TBI and SCI. We collected data at hospital discharge (T1) and at 3 months post-injury (T2). A total of 70 patients, including 49 with TBI and 21 with SCI, with a mean age of 56 years (±21.1, ±17.9) were included. Almost a third of participants with TBI (33%) and SCI (29%) had a moderate average pain intensity at T1, and most experienced mild average pain intensity at T2. At T1, 80% of participants used opioids, whereas at T2, 26% of participants with TBI and 53% of those with SCI did. The main co-analgesic used was acetaminophen, with 78% and 17% for participants with TBI and 81% and 40% for participants with SCI at T1 and T2. The most common non-pharmacological strategy in participants with TBI was rest at T1 (45%) and T2 (32%), and comfortable positioning in participants with SCI at both timepoints (81% and 53%). The two most frequent adverse effects of opioids in both populations at T1 and T2 were drowsiness (35% vs. 43%; 10% vs. 13%) and constipation (27% vs. 38%; 7% vs. 20%). Opioids remain the most widely used pain management strategy in neurotrauma. Promoting a judicious use of opioids, combined with other strategies, could help patients with neurotrauma achieve adequate and safe pain relief.

## Introduction

Every year worldwide, more than 50 million individuals are estimated to sustain a traumatic brain injury (TBI) and more than 27 million a spinal cord injury (SCI).^1^ TBI and SCI are among the leading causes of disability across the globe.^2,3^ Pain is one of the most frequently reported complaints^4–6^ among patients with neurotrauma, and it can hamper recovery during the acute phase as well as daily functioning and quality of life for several years.^7–11^

Given the high prevalence of pain following neurotrauma, opioids are often considered necessary during recovery.^12,13^ Studies show that half of severe patients with TBI receive opioids at discharge, while 30% are still using opioids 1 year later.^14,15^ Additionally, 55% of patients with SCI trauma were prescribed one or more opioids in the first year after hospitalization,^16^ and a similar proportion filled more than one opioid prescription during the third year post-injury.^17^

To minimize the use of opioids and their potential side effects, clinical practice guidelines on the treatment of patients with TBI and SCI recommend using co-analgesia and non-pharmacological strategies.^18–20^ Limited research data are available regarding the use of these strategies in patients with TBI and SCI. Furthermore, little is known about the adverse effects of opioids in the context of neurotrauma to date. We aimed to examine the use of pharmacological and non-pharmacological pain management strategies and to describe the adverse effects of analgesics, particularly opioids, in patients with TBI and SCI with acute and chronic pain.

## Methods

### Study design

We conducted a descriptive longitudinal study in patients with neurotrauma. This study is reported according to the Strengthening the Reporting of Observational Studies in Epidemiology Statement (Supplementary Data S1).^21^

### Setting

The study took place in a Level I trauma center in Canada and in rehabilitation centers or patients’ home. We collected data between December 2020 and July 2022 from consecutive admitted patients with TBI and SCI in the week prior to their discharge from the hospital trauma unit (acute pain: T1) and at 3 months ± 1 week post-injury (chronic pain: T2).^22^ Research Ethics Board approval (CHU de Québec-Université Laval, No. 2021–5513) was granted for this study.

### Eligibility

We included patients with primary diagnosis of mild, moderate, or severe TBI,^23^ or SCI classified as A, B, C, or D on the American Spinal Injury Association Impairment Scale (AIS),^24^ hospitalized for at least 3 days to gain sufficient insight into pain management practices. Participants had to be over 18 years old and speak French. Patients with a psychiatric condition or severe neurocognitive disorder were excluded. Patients could have had concomitant TBI and SCI as well as other injuries, but we excluded patients who underwent surgery for non-neurotraumatic reasons, as we aimed to focus on pain related to neurological injuries.

### Data collection

Eligible patients were identified by the trauma program administrative agent and then met by a member of the research team to obtain informed consent. Given that both target populations often have physical conditions that can prevent them from writing, and that patients with TBI may have cognitive impairment, the questionnaires were administered in-person by a member of the research team at the trauma center (T1) and by telephone after discharge (T2). Family members actively involved in the care of patients with TBI were asked to assist them in completing questionnaires when they had attention and memory problems documented in their medical records, a method that has been shown to provide an adequate estimate of patients’ self-reported pain.^25,26^

### Variables and measures

Sociodemographic and clinical data, as well as data on analgesics administered over the last 3 days prior to hospital discharge, were collected from the patients’ medical records at T1. Participants were asked to describe prescribed or self-administered analgesics they were taking at 3 months and to specify their opioid dosage during the last 3 days at T2.

The *Brief Pain Inventory* (BPI) was used to indicate the most painful areas by circling body parts on a diagram, to measure pain intensity (0: No pain and 10: Most intense pain you can imagine), and to assess the extent of pain interference with daily activities (0: Does not interfere to 10: Completely interferes) over the last 7 days.^27^ The score obtained indicates the severity of pain intensity and interference (1–3: mild; 4–6/10: moderate; 7–10/10: severe).^28–31^ Pain interference with activities was assessed at T2 only, as many of the items from this subscale are not adapted for patients with neurotrauma early after their injury.

The *Neuropathic Pain Symptom Inventory* (NPSI) was used to assess the presence of different neuropathic pain symptoms over the last 24 h. This questionnaire consists of 10 items evaluating five dimensions of neuropathic pain using a 0–10 numerical rating scale.^32^ It also includes two temporal items to describe the frequency of symptoms. The NPSI total score ranges from 0 to 100.^32^

The *Non-Pharmacological Pain Management Strategies Questionnaire* assessed the non-pharmacological strategies used by patients and their perceived level of effectiveness for pain relief during the hospitalization and up to 3 months post-injury. It includes 11 items, each indicating the strategy’s level of effectiveness using a 4-point Likert scale (not effective to very effective).^33^

The *Questionnaire on Opioid-Related Adverse Effects* was used to describe adverse effects in patients using opioids for pain relief over the last 3 days. This tool was adapted from the Opioid-Related Symptom Distress Scale,^34^ a 4-point Likert scale (never to very often) that evaluates three distress dimensions (frequency, intensity, distress) for 12 symptoms, by a group of experts from the Quebec Pain Registry^35^ and the Canadian Neuropathic Pain Database.^38^

Opioid misuse was also assessed using the 8-item *Opioid Compliance Checklist* (OCC) questionnaire.^37^ A positive response to any of the items indicates the likelihood of opioid misuse.^37^ Finally, as cannabis can be used for pain relief, the *Cannabis Abuse Screening Test* (CAST) was used to identify the problematic use of cannabis.^38^ This tool includes six items to evaluate habits, behavior, and the effects of cannabis on the user.^39^ The OCC and CAST were administered at T2 only, as the OCC was designed to assess opioid misuse in the context of chronic pain,^37^ and cannabis was not prescribed during the study. All measurement tools have been validated in French^40–44^ except those on the non-pharmacological strategies and adverse effects of opioids, which consist solely of an item list.

### Data analysis

Statistical analyses were performed using SPSS (Statistical Package for Social Sciences v27; IBM, Armonk, NY). We calculated means and standard deviations for continuous variables measured at both timepoints and neurotrauma populations. Categorical variables were converted into frequencies and percentages (%). Opioid intakes were quantified in oral morphine equivalent doses per day (MEDD).^40^

## Results

### Participant characteristics

**FIG. 1.**
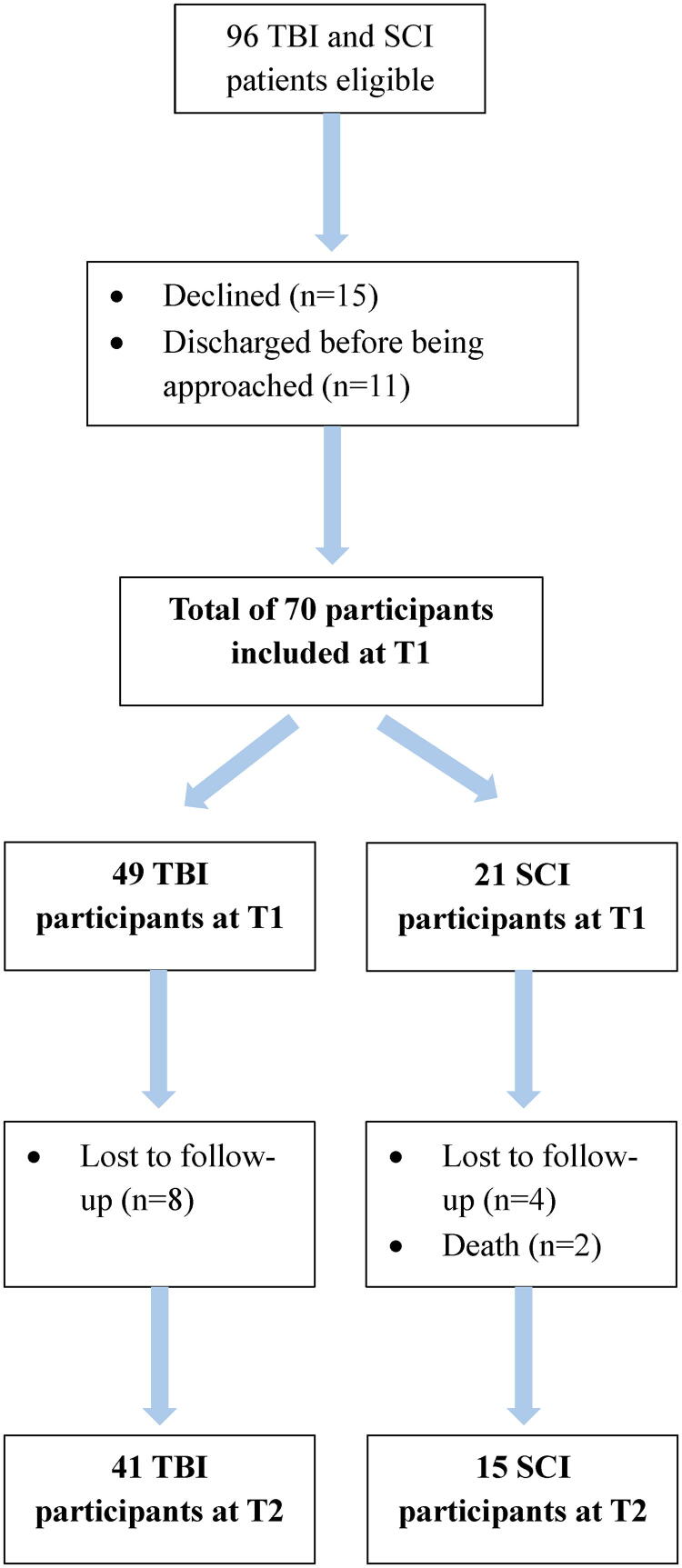
Flow diagram of included participants at T1 and T2.

During the recruitment period, 96 patients were eligible, 70 (73%) agreed to participate, 15 (16%) declined, and 11 (12%) were discharged from the hospital before being approached to participate (Fig. 1). The initial sample of 70 participants consisted of 49 patients with TBI (70%) and 21 patients with SCI (30%).

The mean age of the sample was 56 years (±21.1, ±17.9), with similar results for both studied populations. There were more men included in participants with TBI and SCI (63% vs. 86%; Table 1). Among those with a TBI, most had mild TBI (47%), while most with a SCI were tetraplegic (76%) and scored D on the AIS (43%). Despite attrition, participants’ characteristics were similar at T2 (Supplementary Data S2).

**Table 1.** Sociodemographic and Clinical Characteristics of Participants

Characteristics	TBI (*N* = 49)	SCI (*N* = 21)
Age, mean (SD)	56 (21.1)	56 (17.9)
Male, *N* (%)	31 (63)	18 (86)
Ethnicity, *N* (%)		
Indigenous North American	1 (2)	0
White North American	47 (96)	20 (95)
Latin, Central, and South America	0	1 (5)
Highest level of education completed, *N* (%)		
Elementary	21 (43)	4 (19)
High school	12 (25)	10 (48)
College or professional diploma	15 (31)	7 (33)
TBI, *N* (%)		
Severe	10 (20)	1 (5)
Moderate	16 (33)	0
Mild	23 (47)	6 (29)
SCI, *N* (%)		
Tetraplegic	—	16 (76)
Paraplegic	—	5 (24)
ASI A	—	3 (14)
ASI B	—	2 (10)
ASI C	—	7 (33)
ASI D	—	9 (43)
Other injuries,^a,b^ *N* (%)	25 (51)	9 (43)
Mechanisms of injury, *N* (%)		
Fall	26 (53)	9 (43)
Motor vehicle collision	14 (29)	8 (38)
Pedestrian	3 (6)	0
Sport	1 (2)	2 (10)
Other	4 (8)	2 (10)
Surgery required, *N* (%)	14 (29)	19 (91)
Hospital length of stay, median (range)	14 (3–52)	22 (6–36)
Opioid use before hospitalization, *N* (%)	3 (6)	0

^a^ Other injuries in participants with TBI in descending order: facial and cranium fracture, thoracic injury, shoulder injury, limb fracture, and abdominal injury.

^b^ Other injuries in participants with SCI in descending order: mild TBI, spine fracture, and limb fracture.

ASI, American Spinal Injury Association Impairment Scale; SCI, spinal cord injury; SD, standard deviation; TBI, traumatic brain injury.

### Pain in the acute and chronic phases

**FIG. 2.**
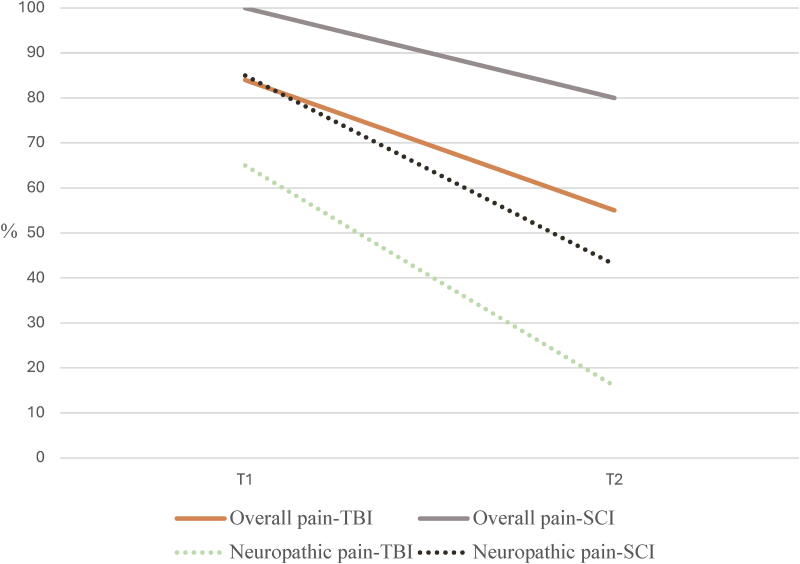
Proportions of participants with TBI and SCI with any type of pain and with neuropathic pain at T1 and T2. SCI, spinal cord injury; TBI, traumatic brain injury.

**FIG. 3.**
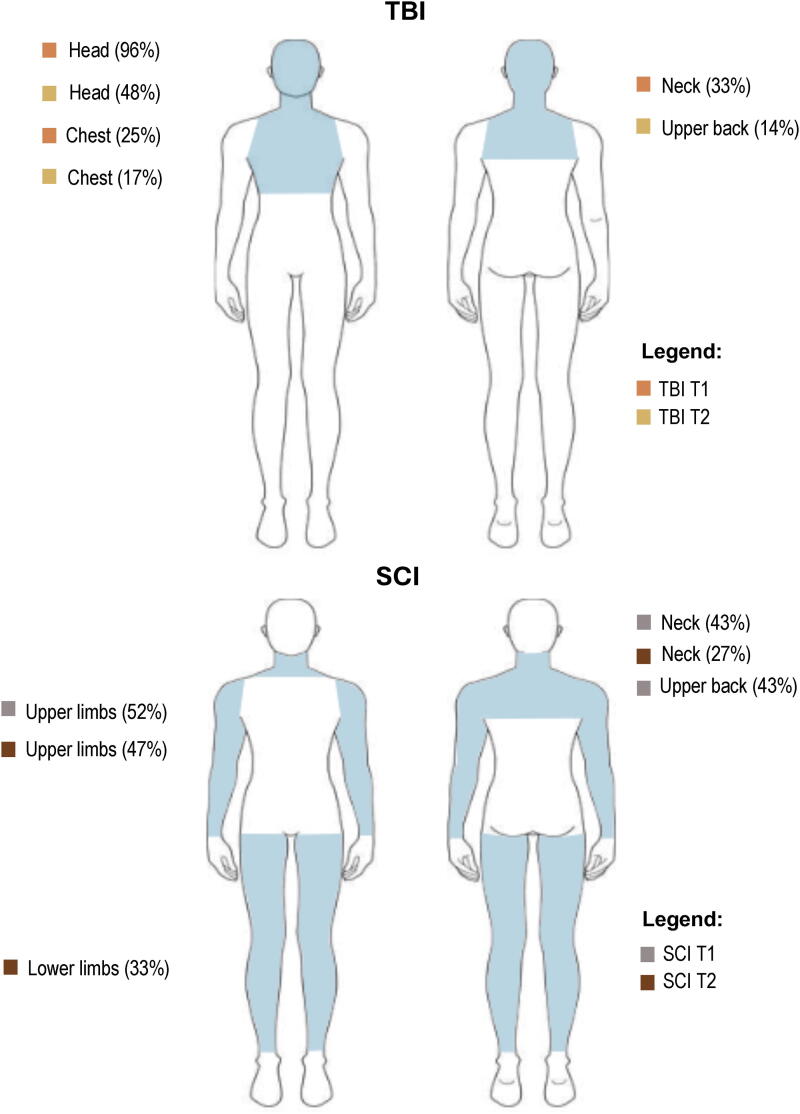
Most common sites of pain in participants with TBI and SCI at T1 and T2. SCI, spinal cord injury; TBI, traumatic brain injury.

In the acute phase (T1), 84% of patients with TBI and 100% of patients with SCI reported pain, whereas in the chronic phase (T2), 55% and 80% reported pain, respectively (Fig. 2). Among those with pain, 65% of participants with TBI and 85% of participants with SCI had neuropathic pain at T1, while 16% of the former and 43% of the latter experienced this type of pain at T2. At both timepoints, the primary site of pain was the head (96% and 48%) in participants with TBI and the upper limbs in those with SCI (52% and 47%; Fig. 3).

**FIG. 4.**
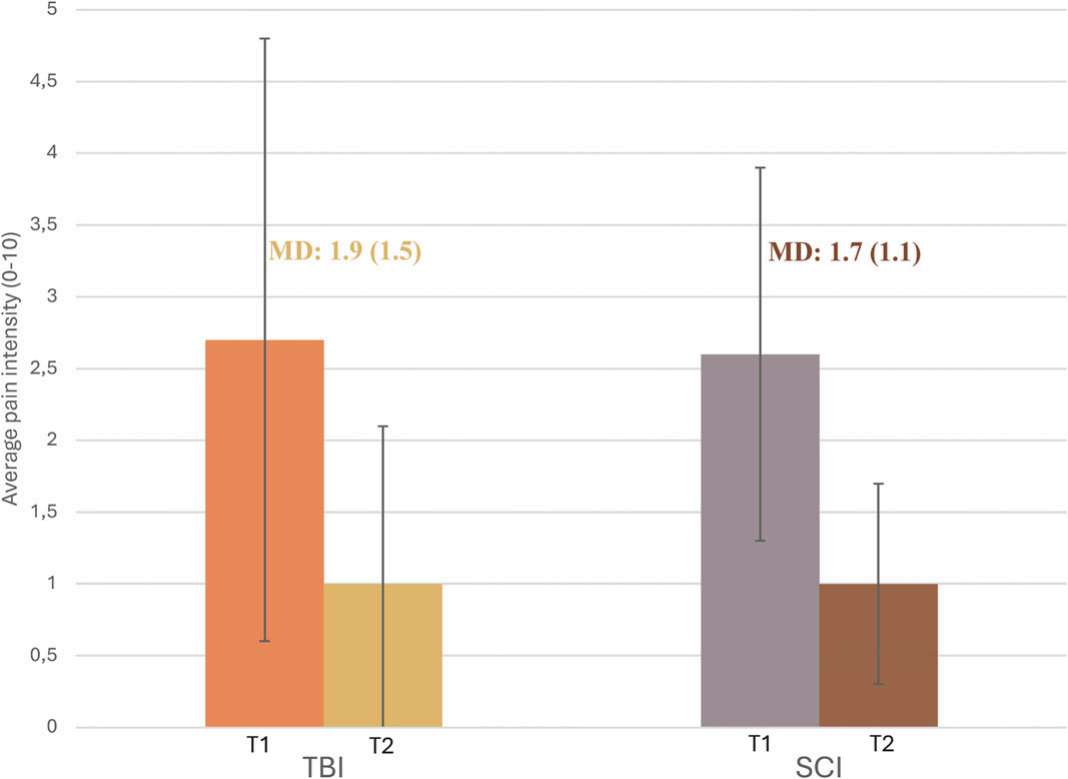
Mean average pain intensity in participants with TBI and SCI at T1 and T2, with the mean difference between timepoints. SCI, spinal cord injury; TBI, traumatic brain injury.

**FIG. 5.**
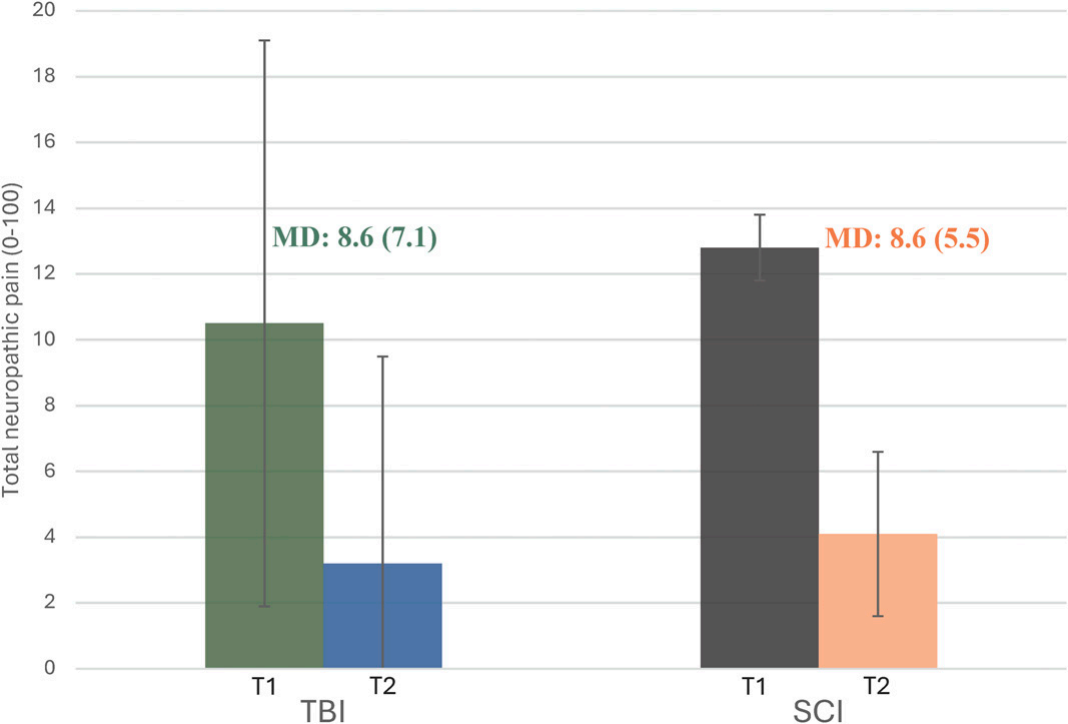
Mean total neuropathic pain in participants with TBI and SCI at T1 and T2, with the mean difference between timepoints. SCI, spinal cord injury; TBI, traumatic brain injury.

Participants with TBI and SCI experienced mild average pain intensity at T1 and T2, but one-third of them had moderate average pain intensity at T1 (33% vs. 29%; Table 2). The worst pain intensity was moderate in participants with TBI and SCI at T1 and mild at T2, but still moderate in one-third at the last timepoint (32% vs. 27%). Mean scores for average pain intensity (Fig. 4) and total neuropathic pain (Fig. 5) improved in all participants from whom data were collected at the two timepoints. Both the TBI and SCI groups reported mild pain interference with activities at T2.

**Table 2.** Pain in Participants with Traumatic Brain Injury and Spinal Cord Injury at T1 and T2

	T1		T2	
	TBI (*N* = 49)	SCI (*N* = 21)	TBI (*N* = 41)	SCI (*N* = 15)
Average pain intensity, *N* (%)				
No pain	8 (16)	0	19 (45)	3 (20)
Mild (1–3/10)	24 (49)	15 (71)	22 (52)	12 (80)
Moderate (4–6/10)	16 (33)	6 (29)	1 (2)	0
Severe (7–10/10)	1 (2)	0	0	0
Mean (0–10) (SD)	2.7 (2.1)	2.6 (1.3)	1.0 (1.1)	1.0 (0.7)
Worst pain intensity, *N* (%)				
No pain	—	—	11 (27)	1 (7)
Mild (1–3/10)	11 (22)	3 (14)	16 (39)	10 (67)
Moderate (4–6/10)	16 (33)	10 (48)	13 (32)	4 (27)
Severe (7–10/10)	22 (45)	8 (38)	1 (2)	0
Mean (0–10) (SD)	6.1 (2.6)	6.0 (2.0)	2.6 (2.0)	2.9 (1.4)
Overall pain interference with activities, *N* (%)				
No pain interference	—	—	20 (49)	12 (80)
Mild (1–3/10)	—	—	18 (44)	2 (13)
Moderate (4–6/10)	—	—	2 (5)	1 (7)
Severe (7–10/10)	—	—	1 (2)	0
Mean (0–10) (SD)			0.7 (1.0)	0.3 (0.4)
No neuropathic pain	16 (35)	3 (15)	32 (84)^a^	8 (57)
Total neuropathic pain score (0–100), mean (SD)	10.5 (8.6)	12.8 (6.3)	3.2 (4.0)	4.1 (2.5)
Burning (superficial) spontaneous pain (0–10)	0.5 (1.7)	2.1 (2.7)	0.5 (1.3)	1.2 (1.4)
Pressing (deep) spontaneous pain) (0–10)	3.1 (2.9)	1.8 (2.3)	0.6 (1.0)	0.2 (0.5)
Paroxysmal pain (0–10)	1.1 (2.0)	3.7 (2.6)	0.5 (1.0)	1.7 (1.8)
Evoked pain (0–10)	5.6 (4.3)	4.7 (4.5)	1.6 (2.4)	1.0 (2.1)
Paresthesia/dysesthesia (0–10)	0.1 (0.4)	4.2 (2.6)	0.1 (0.3)	1.1 (1.0)
Frequency for the spontaneous pain, *n* (%)				
Consistently	9 (18)	6 (29)	1 (3)	0
8–12 h/day	18 (38)	10 (48)	8 (20)	6 (40)
4–7 h/day	17 (35)	1 (5)	10 (25)	3 (20)
1–3 h/day	2 (4.2)	3 (14)	7 (18)	4 (27)
<1 h	2 (4.2)	1 (5)	14 (35)	2 (13)
Frequency for the paroxysmal pain, *n* (%)				
>20	2 (4)	7 (33)	0	0
11–20/day	10 (21)	8 (38)	4 (10)	3 (20)
6–10/day	3 (6)	1 (5)	2 (5)	3 (20)
1–5/day	5 (10)	1 (5)	7 (18)	3 (20)
None	28 (58)	4 (19)	27 (68)	6 (40)

^a^ Data were available from only 38 out of 41 patients.

SCI, spinal cord injury; SD, standard deviation; TBI, traumatic brain injury.

### Pain management strategies

#### Pharmacological

**FIG. 6.**
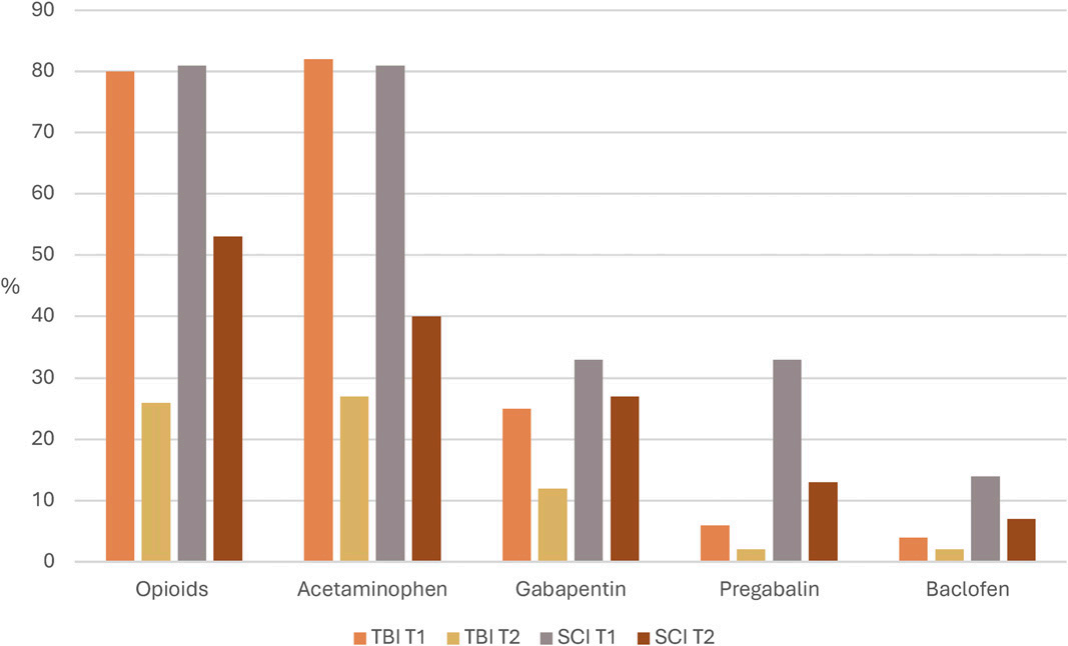
Proportions of analgesics used by participants with TBI and SCI at T1 and T2. SCI, spinal cord injury; TBI, traumatic brain injury.

About 80% of participants with TBI and SCI used opioids at T1, and a quarter of participants with TBI (25%) and just over half of those with SCI (56%) used this analgesic at T2 (Fig. 6). Participants with TBI took a mean MEDD of 7.1 mg (±11.3) and participants with SCI a mean MEDD of 12.1 (±17.7) at T1. Mean MEDD was slightly higher in participants with TBI at T2 (9.1 mg ± 10.4) and was lower in participants with SCI (7.3 mg ± 9.4). The most used co-analgesic at T1 for participants with TBI and SCI was acetaminophen (78% vs. 81%). However, three-thirds of participants with SCI used gabapentinoids at this timepoint (67%). At T2, acetaminophen and gabapentinoids were still the most used co-analgesics, although more so in participants with SCI than participants with TBI (40% vs. 17%; 40% vs. 14%).

#### Non-pharmacological

Rest was the most used non-pharmacological strategy for participants with TBI (45%) and comfortable positioning for participants with SCI (81%) at T1 (Tables 3 and 4). These strategies were also perceived as the most highly effective in both populations. The second most used strategy was comfortable positioning in participants with TBI (43%), while they were distraction and relaxation in participants with SCI (24%), although only the latter was perceived as highly effective by some participants (20%). At T2, rest was still the most used strategy by participants with TBI (32%) and comfortable positioning by participants with SCI (53%). Both groups reported, massage and rest as being the most effective at 3 months post-injury.

**Table 3.** Non-Pharmacological Strategies Used by Participants with Traumatic Brain Injury at T1 and T2

	T1 (*N* = 49)			T2 (*N* = 41)		
Strategies	*N* (%)	Low efficacy *N* (%)^*a*^	High efficacy *N* (%)^*a*^	*N* (%)	Low efficacy *N* (%)^*a*^	High efficacy *N* (%)^*a*^
Massage	1 (2)	1 (100)	—	2 (5)	1 (50)	1 (50)
Comfortable positioning	21 (43)	16 (76)	5 (24)	10 (24)	10 (100)	—
Mental imagery	1 (2)	1 (100)	—	1 (2)	1 (100)	—
Distraction	6 (12)	6 (100)	—	3 (7)	3 (100)	—
Relaxation	9 (18)	8 (89)	1 (11)	5 (12)	5 (100)	—
Rest	22 (45)	14 (64)	6 (27)	13 (32)	10 (77)	2 (15)
Breathing techniques	2 (4)	1 (50)	—	2 (5)	2 (100)	—
Touch	1 (2)	1 (100)	—	—	—	—
Creation of a comfortable environment	1 (2)	1 (100)	—	2 (5)	2 (100)	—
Others^b^	7 (14)	5 (71)	2 (29)	8 (20)	5 (63)	3 (38)

Strategies in blue were the most frequently used and those most often perceived as highly effective at T1 and T2.

^a^ Proportions calculated based on the number of participants who used the strategies.

^b^ Others included thermotherapy (cold and heat).

**Table 4.** Non-Pharmacological Strategies Used by Participants with Spinal Cord Injury at T1 and T2

	T1 (*N* = 21)			T2 (*N* = 15)		
Strategies	*N* (%)	Low efficacy *N* (%)^*a*^	High efficacy *N* (%)^*a*^	*N* (%)	Low efficacy *N* (%)^*a*^	High efficacy *N* (%)^*a*^
Massage	0	—	—	3 (20)	2 (67)	1 (33)
Physical positioning	17 (81)	11 (65)	5 (29)	8 (53)	7 (88)	1 (13)
Mental imagery	1 (5)	—	1 (100)	0	—	—
Distraction	5 (24)	5 (100)	—	5 (33)	5 (100)	—
Relaxation	5 (24)	4 (80)	1 (20)	3 (20)	3 (100)	—
Sleep	3 (14)	3 (100)	—	1 (7)	1 (100)	—
Breathing techniques	—	—	—	0	—	—
Touch	—	—	—	0	—	—
Creation of a comfortable environment	—	—	—	3 (20)	3 (100)	—
Others^b^	1 (5)	1 (100)	—	1 (7)	1 (100)	—

Strategies in blue were the most frequently used and those most often perceived as highly effective at T1 and T2.

^a^ Proportions calculated based on the number of participants who used the strategy.

^b^ Others included thermotherapy (cold and heat).

#### Adverse effects of opioids

**FIG. 7.**
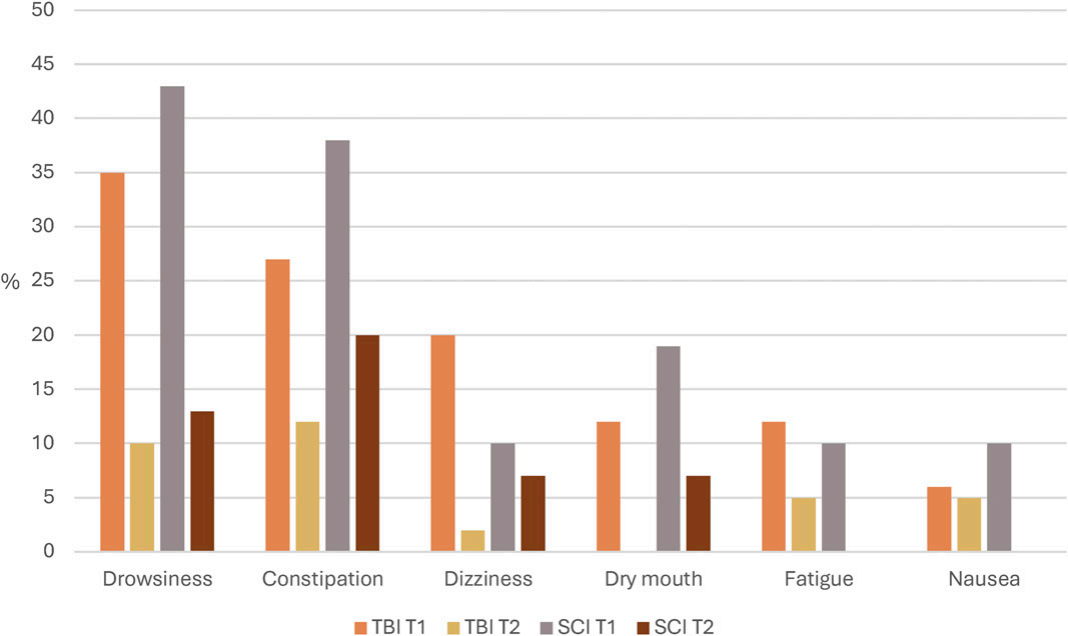
Proportions of the most common adverse effects of opioids in participants with TBI and SCI at T1 and T2. SCI, spinal cord injury; TBI, traumatic brain injury.

During the hospitalization, the three common adverse effects of opioids use in participants with TBI were drowsiness (35%), constipation (27%), and dizziness/lightheadedness (20%), while drowsiness (43%) and constipation (38%) were the most observed in participants with SCI (Fig. 7). The intensity and distress associated with these adverse effects were mostly mild to moderate (Supplementary Data S3 and S4). At 3 months, the two main adverse effects for both studied neurotrauma populations were drowsiness (10% vs. 13%) and constipation (12% vs. 20%). The intensity and distress reported were generally mild. As for patients still using opioids at 3 months and likely to develop opioid misuse (*n* = 19), 32% were in the TBI group and none in the SCI group. Likewise, among the participants using cannabis at T2 (TBI, *n* = 14; SCI, *n* = 2), 63% were at low risk, 25% at moderate risk, and 13% at high risk of dependence.

## Discussion

In our study, we described pain management strategies used in a cohort of patients with neurotrauma with acute and chronic pain and the adverse effects they experienced regarding opioid use. One-third of patients with TBI and SCI had average pain intensity reaching moderate intensity before being discharged, while the majority reached this threshold for the worst pain experienced at 3 months. Opioids and acetaminophen were the most frequently used pharmacological strategies. The most used non-pharmacological strategies were comfortable positioning, rest, and distraction. Around one-third of the patients experienced mild to moderate form of opioid-related adverse effects, with drowsiness and constipation being the most reported.

In our study, 80% of the patients used opioids before discharge from the hospital. This is a larger proportion than reported in prior research performed in patients with isolated TBI, with just over half of them using opioids at discharge.^14^ One possible explanation for this difference is that 50% of participants with TBI also had other injuries in our study, which could also be major sources of pain. Likewise, acute pain has been reported in 60% of patients with TBI in previous studies,undefined,^45^ whereas it was observed in 84% of our cohort. Regarding patients with SCI, most research data described significant opioid use in the acute and chronic phase, and a trend toward greater long-term opioid use has been reported in this population compared to patients with TBI.^14,16,17^ The diversity of pain sources in patients with SCI could explain this greater opioid consumption. In this respect, although opioids are not indicated for the treatment of neuropathic pain in patients with SCI,^19^ isolating this type of pain from others can sometimes be complex.

The opioid dose used by participants in our study was lower than that associated with a risk of overdose in those naïve to this analgesic (<20 MEDD).^46^ Similarly, participants used three times less opioids than a cohort of patients with orthopedic injuries previously studied.^47^ However, despite the limited opioid use in our study in patients with TBI, nearly one-third of them were at risk of opioid misuse, while the same proportion of cannabis users was at moderate to high risk of dependence. Given the brain damage associated with TBI, some of these patients can develop behavioral disorders, leading to impaired judgment regarding substance use.^48^ It may therefore be important to provide additional support to patients with TBI using opioids as soon as they are discharged from the hospital, as well as to those using cannabis.

As recommended by clinical practice guidelines,^18–20^ co-analgesia, particularly acetaminophen, was widely used in participants with TBI and SCI during the acute phase, although it was not used in 20% of them. Similarly, the use of acetaminophen greatly decreased and was even lower than that of opioids during the transition to the chronic phase. The same was true of gabapentinoids, particularly in patients with SCI, in whom this agent is recommended as first-line treatment for neuropathic pain.^19^ The decrease in the ratio between opioid use and co-analgesia at 3 months post-injury was not observed in a prior study on orthopedic trauma.^47^ One explanation may be that patients with neurotrauma experienced less pain than those who sustained orthopedic trauma in the long term, as shown by lower mean scores for pain.^47^ In addition, acetaminophen was shown to be an effective treatment for musculoskeletal pain,^49,50^ whereas this analgesic is better known for its synergistic effect with other agents in the treatment of neuropathic pain.^51^ Nevertheless, the results obtained in our study demonstrate the possibility of optimizing co-analgesia in neurotrauma, particularly in the acute phase and in patients experiencing pain flare-ups or neuropathic pain along the continuum of care.

For non-pharmacological strategies, although recommended in guidelines for pain management following a TBI or a SCI, they are still associated with low-level evidence in these populations.^19,52–54^ The lack of evidence likely explains the poor implementation of these modalities during patients’ hospitalization. This in turn could have led to the limited use by patients at 3 months post-injury, considering there was little exposure to these strategies while receiving acute care. Previous studies in patients with SCI with chronic pain showed that many strategies such as massage, body position adjustment, relaxation therapy, and exercise were deemed effective by three-quarters of patients by 1 year post-injury.^55,56^ The discrepancy between our results and those of previous research could be explained by the fact that patients experiencing chronic pain for a longer time may seek non-pharmacological strategies more frequently.^57^ Therefore, such strategies should not be completely overlooked in the care trajectory of patients with neurotrauma.

Guidelines on opioid therapy underscore the substantial risks associated with the use of these analgesics.^18,58–60^ A study of orthopedic injury patients revealed that a larger proportion experienced adverse events affecting the central nervous system, particularly shortly before discharge from the hospital, than the patients in our study.^47^ The differences in adverse events occurring early after injury could be explained by greater opioid consumption in patients with orthopedic injuries^47^ than in patients with TBI and SCI. Although the patients in our study reported mild to moderate opioid adverse effects, the impact on their health is worthy of attention. Drowsiness, dizziness, and fatigue are common symptoms following TBI,^61^ and taking opioids may exacerbate these. The same applies to constipation after SCI.^62^

### Strengths and limitations

Our study provides an overview of pain management strategies used, including opioids and their adverse effects, in patients with neurotrauma, areas that have been little explored to date in this population. Consequently, our results provide relevant information to health care professionals and researchers on potential avenues for improvement to offer optimal and safe pain management in patients with TBI and SCI.

Our study has also some limitations. First, we only collected data at the early stage of chronic pain onset, which limits the perspective on pain relief when individuals with neurotrauma return to their daily activities and on adverse effects that might occur with prolonged opioid use. However, this study still made it possible to identify avenues for improvement in pain management strategies to be optimized. Second, opioid use at 3 months, as well as adverse effects of this analgesic at both measurement times, was self-reported or derived from family members’ responses. Therefore, it is possible that these outcomes were not accurately portrayed. However, the fact that participants used similar doses of opioids at 3 months and prior to hospital discharge without experiencing more adverse effects suggests that the data are representative. Third, our study was carried out in a single setting, limiting the external validity of the results. In addition, our sample consisted mainly of middle-aged White males with mild TBI or incomplete SCI. Therefore, how our data compared to that from patients with TBI and SCI with different social diversity criteria and more severe forms of injury remain to be verified.

## Conclusions

Our study highlighted that many patients with TBI and SCI used opioids in the presence of acute pain and at the chronic pain onset, and yet many experienced significant pain. However, opioids should still be used with caution, considering that they can cause adverse effects and carry a risk of misuse, particularly in patients with TBI. The results of this study demonstrated the possibility of optimizing co-analgesia and the use of non-pharmacological strategies in the continuum of care of patients with TBI and SCI. Multidisciplinary pain management protocols that take these areas of improvement into account and that can be deployed during hospitalization and in the first few months after neurotrauma may help promote judicious use of opioids and other pain management strategies in neurotrauma populations.

## Transparency, Rigor, and Reproducibility Statement

This study and its analysis plan were not formally registered because of the nature of the design used (descriptive only). A sample of 80 patients (60 with TBI and 20 with SCI) was targeted in the initial protocol submitted to scientific and ethical review, based on the estimation of the number of potentially eligible patients according to admissions/year of both populations in the trauma center where the participants were recruited. However, given the COVID-19 pandemic and the decrease in trauma admissions, particularly at the beginning of this period, we extended the data collection to one and a half years rather than 1 year. A total of 96 patients were eligible to participate in the study from December 2020 to July 2022 and 70 ultimately agreed to participate, including 49 patients with TBI and 21 patients with SCI. In total, 70 participants completed the questionnaires at T1, and 56 participants completed them at T2. Data analysis included responses provided by each of these participants. Eight TBI participants and six SCI participants were lost to follow-up, including two in the latter group who died from causes unrelated to their injury before the end of the study. Reasons for incomplete assessments include inability to contact the participant or a clinical condition leading to inability to complete the questionnaires. Data were analyzed using SPSS® version 29 software (Statistical Package for the Social Sciences version 29; IBM). Anonymized data from this study are not available in a public repository. Anonymized data from this study will be made available (according to institutional institutional review board standards) by sending an email to the corresponding author as of April 30, 2025. No analytical codes are associated with this study. The authors agree or have agreed to publish the article using the Mary Ann Liebert Inc. Open Access format under a Creative Commons/CC-BY license.

## Abbreviations Used

AIS: American Spinal Injury Association Impairment Scale
BPI: Brief Pain Inventory
CAST: Cannabis Abuse Screening Test
CHU: Centre hospitalier universitaire
MD: Mean difference
MEDD: Morphine Equivalent Doses per Day
NPSI: Neuropathic Pain Symptom Inventory
OCC: Opioid Compliance Checklist
SCI: Spinal Cord Injury
SD: Standard deviation
SPSS: Statistical Package for Social Sciences
TBI: Traumatic Brain Injury
