# Supplementary material for: Pain Management Strategies and Adverse Effects of Opioids in Patients with Neurotrauma with Acute and Chronic Pain
Source: Neurotrauma Rep. 2025 Aug 19;6(1):686–99. doi: 10.1177/08977151251365585 (PMC12413256; doi:10.1177/08977151251365585)
Supplement: Supplementary Data S2 [file 08977151251365585_supplementary_data_s2.docx]

**Supplement 2.** **Sociodemographic and clinical characteristics of participants at T1 and T2**

|  | **T1** | | **T2** | |
| --- | --- | --- | --- | --- |
|  | **TBI (N=49)** | **SCI (N=21)** | **TBI (N=41)** | **SCI (N=15)** |
| Age, Mean (SD) | 56 (21.1) | 56.0 (17.9) | 54 (20.3) | 55 (18.0) |
| Male, N (%) | 31 (63) | 18 (86) | 27 (64) | 13 (87) |
| Ethnicity, N (%) |  |  |  |  |
| Indigenous North American | 1 (2) | 0 | 1 (2) | 0 |
| White North American | 47 (96) | 20 (95) | 40 (95) | 15 (100) |
| Latin, Central and South America | 0 | 1 (5) | 0 | 0 |
| Highest level of education completed, N (%) |  |  |  |  |
| Elementary | 21 (43) | 4 (19) | 17 (41) | 2 (13) |
| High school | 12 (25) | 10 (48) | 11 (26) | 8 (53) |
| College or professional diploma | 15 (31) | 7 (33) | 13 (31) | 5 (33) |
| TBI, N (%) |  |  |  |  |
| Severe | 10 (20) | 1 (5) | 9 (21) | 0 |
| Moderate | 16 (33) | 0 | 13 (31) | 0 |
| Mild | 23 (47) | 6 (29) | 17 (41) | 4 (27) |
| Spinal cord injury, N (%) |  |  |  |  |
| Tetraplegic  Paraplegic  ASI A | -  -  - | 16 (76)  5 (24)  3 (14) | -  -  - | 11 (73)  4 (27)  1 (7) |
| ASI B | - | 2 (10) | - | 2 (13) |
| ASI C | - | 7 (33) | - | 5 (33) |
| ASI D | - | 9 (43) | - | 7 (47) |
| Other injuries*^+^, N (%) | 25 (51) | 9 (43) | 23 (55) | 6 (40) |
| Surgery required, N (%) | 14 (29) | 19 (91) | 11 (27) | 14 (93) |
| Mechanisms of injury, N (%) |  |  |  |  |
| Fall | 26 (53) | 9 (43) | 22 (52) | 4 (27) |
| Motor vehicle collision | 14 (29) | 8 (38) | 13 (31) | 7 (47) |
| Pedestrian | 3 (6.) | 0 | 3 (7.) | 0 |
| Sport | 1 (2) | 2 (10) | 0 | 2 (13) |
| Other | 4 (8) | 2 (10) | 3 (7) | 2 (13) |
| Opioid use before hospitalization, N (%) | 3 (6) | 0 | 3 (7) | 0 |

^*^Other injuries in TBI participants in descending order: facial and cranium fracture, thoracic injury, shoulder injury, limb fracture, abdominal injury

^+^Other injuries in SCI participants in descending order: mild TBI, spine fracture, limb fracture

SCI: Spinal cord injury; SD: Standard deviation, TBI: Traumatic brain injury
