# Supplementary material for: Pain Management Strategies and Adverse Effects of Opioids in Patients with Neurotrauma with Acute and Chronic Pain
Source: Neurotrauma Rep. 2025 Aug 19;6(1):686–99. doi: 10.1177/08977151251365585 (PMC12413256; doi:10.1177/08977151251365585)
Supplement: Supplementary Data S3 [file 08977151251365585_supplementary_data_s3.docx]

**Supplement 3.** **Adverse effects of opioids in TBI participants at T1 and T2**

|  | **T1**  **(N=49)** | | | **T2**  **(N=41)** | | |
| --- | --- | --- | --- | --- | --- | --- |
| **Adverse effects** | **Frequency** | **Intensity** | **Distress*** | **Frequency** | **Intensity** | **Distress*** |
| Dizziness/lightheadedness, N (%) | 10 (20) | Mild: 5 (50)  Moderate: 5 (50) | Mild: 5 (50)  Moderate: 4 (40) | 1 (2) | Mild: 1 (100) | Mild: 1 (100) |
| Drowsiness, N (%) | 17 (35) | Mild: 15 (88) | Mild: 3 (18) | 4 (10) | Mild: 4 (100) | Mild: 2 (50) |
| Confusion, N (%) | - | Moderate: 2 (12)  - | Moderate: (6)  - | - | - | - |
| Nausea, N (%) | 3 (6) | Mild: 1 (33) | Moderate: 1 (33) | - | - | - |
| Vomiting, N (%) | 1 (2) | Moderate : 2 (67)  Moderate: 1 (100) | Mild: 1 (100) | - | - | - |
| Memory loss, N (%) | 1 (2) | Mild : 1 (100) | Mild: 1 (100) | 1 (2) | Mild : 1 (100) | Mild : 1 (100) |
| Dry mouth, N (%) | 6 (12) | Mild: 2 (33) | Mild: 4 (67) | - | - | - |
| Itching, N (%) | - | Moderate: 4 (67)  - | Moderate: 2 (33)  - | 1 (2) | Moderate: 1 (100) | Mild: 1 (100) |
| Abdominal discomfort, N (%) | 2 (4) | Mild: 1 (50) | Mild : 2 (100) | 1 (2) | Mild : 1 (100) | - |
| Constipation, N (%) | 13 (27) | Moderate : 1 (50)  Mild: 8 (62) | Mild: 7 (54) | 5 (12) | Mild: 4 (80) | Mild: 2 (40) |
| Decreased urine flow, N (%) | - | Moderate: 5 (39)  - | Moderate: 2 (15) | - | Moderate: 1 (20)  - | - |
| Fatigue, N (%) | 6 (12) | Mild: 3 (50) | Mild: 3 (50) | 2 (5) | Mild: 1 (50) | Mild : 1 (50) |
| Insomnia, N (%) | - | Moderate: 3 (50)  - | - | - | Moderate: 1 (50)  - | Moderate: 1 (50)  - |
| Swelling, N (%) | - | - | - | - | - | - |
| Weight gain, N (%) | - | - | - | - | - | - |
| Blurred vision, N (%) | - | - | - | - | - | - |
| Decreased libido, N (%) | 1 (2) | Moderate: 1 (100) | Moderate : 1 (100) | 1 (2) | Moderate: 1 (100) | Mild: 1 (100) |
| Hallucinations, N (%) | - | - | - | - | - | - |
| Nightmares, N (%) | - | - | - | - | - | - |

*Some participants reported no distress associated with opioid adverse effects
