# Supplementary material for: Pain Management Strategies and Adverse Effects of Opioids in Patients with Neurotrauma with Acute and Chronic Pain
Source: Neurotrauma Rep. 2025 Aug 19;6(1):686–99. doi: 10.1177/08977151251365585 (PMC12413256; doi:10.1177/08977151251365585)
Supplement: Supplementary Data S4 [file 08977151251365585_supplementary_data_s4.docx]

**Supplement 4.** **Adverse effects of opioids in SCI participants at T1 and T2**

|  | **T1**  **(N=21)** | | | **T2**  **(N=15)** | | |
| --- | --- | --- | --- | --- | --- | --- |
| **Adverse effects** | **Frequency** | **Intensity** | **Distress*** | **Frequency** | **Intensity** | **Distress*** |
| Dizziness/lightheadedness, N (%) | 2 (10) | Mild: 1 (50)  Moderate: 1 (50) | Mild: 1 (50) | 1 (7) | Mild: 1 (100) | - |
| Drowsiness, N (%) | 9 (43) | Mild: 7 (78) | Mild: 3 (18) | 2 (13) | Mild: 2 (100) | - |
| Confusion, N (%) | - | Moderate: 2 (22)  - | Moderate: (6)  - | - | - | - |
| Nausea, N (%) | 2 (10) | Mild: 1 (50) | Mild: 1 (100) | - | - | - |
| Vomiting, N (%) | - | Moderate : 1 (50)  - | - | - | - | - |
| Memory loss, N (%) | - | - | - | - | - | - |
| Dry mouth, N (%) | 4 (19) | Mild: 1 (25) | Mild: 1 (25) | 1 (7) | 1 (100) | - |
| Itching, N (%) | 1 (5) | Moderate: 3 (75)  - | Moderate: 2 (50)  - | - | - | - |
| Abdominal discomfort, N (%) | 1 (5) | Moderate: 1 (100) | Mild : 1 (100) | - | - | - |
| Constipation, N (%) | 8 (38) | Mild: 3 (38) | Mild: 3 (38) | 3 (20) | Mild: 4 (80) | Mild: 2 (40) |
| Decreased urine flow, N (%) | - | Moderate: 5 (63)  - | Moderate: 2 (25) | - | - | - |
| Fatigue, N (%) | 2 (10) | Mild: 3 (100) | Mild: 1 (50) | - | - | - |
| Insomnia, N (%) | - | - | Moderate: 1 (50)  - | - | - | - |
| Swelling, N (%) | - | - | - | - | - | - |
| Weight gain, N (%) | - | - | - | - | - | - |
| Blurred vision, N (%) | - | - | - | - | - | - |
| Decreased libido, N (%) | - | - | - | - | - | - |
| Hallucinations, N (%) | - | - | - | - | - | - |
| Nightmares, N (%) | - | - | - | - | - | - |

*Some participants reported no distress associated with opioid adverse effects
